# Endophenotypes in psychiatric disease: prospects and challenges

**DOI:** 10.1186/s13073-018-0526-5

**Published:** 2018-02-22

**Authors:** William G. Iacono

**Affiliations:** Department of Psychology, University of Minnesota, 75 East River Road, Minneapolis, MN 55455 USA

## Abstract

Endophenotypes, quantitative neurobehavioral traits that index genetic susceptibility for a psychiatric disorder, have been examined in thousands of studies. Nevertheless, they have underexploited potential to provide etiological insights into prognosis, how psychopathology develops, the etiology of comorbidity, and the mechanisms of gene function.

## Utility of endophenotypes

Molecular genetic studies have shown that we can reliably identify genetic variants that are associated with psychiatric disorders using consortium-based, meta-analyzed samples, with pooled samples including the genomes of over 100,000 individuals now becoming realistically attainable. These investigations have shown that mental disorders are polygenic, with thousands of variants contributing to disease liability. Against this backdrop of progress, it is timely to ask whether we can do a better job of isolating genetic variants using endophenotypes.

Endophenotypes are heritable traits that are derived from laboratory measures such as electroencephalographic anomalies, neurocognitive performance deficits, and impaired facial emotion recognition. They appear in both patients and their unaffected relatives. Because they are presumed to be more proximal to the action of genes than clinical diagnoses, they may enable the identification of genetic variants and associated genes using small samples. Originally conceived by Gottesman and Shields [1], endophenotypes were envisioned to be disorder-specific and diagnostically confirmatory.

Here, we discuss challenges to the conventional wisdom that endophenotypes can facilitate gene discovery and discuss recent studies highlighting the novel contributions that endophenotypes are making to our understanding of psychopathology.

## Challenges for the utility of endophenotypes

Because diagnoses are obtained as a routine aspect of clinical treatment and psychopathology research, they are readily obtainable without incurring additional costs. Endophenotype measures, by contrast, typically require intensive laboratory assessment by skilled staff, using expensive and time-consuming procedures that are potentially off-putting to participants. A cost-benefit appraisal suggests that to assist gene discovery, endophenotypes must have properties that render their application cost-efficient. Ideally, they should be genetically simpler than clinical phenotypes, thus making their genetic architecture more easily discernible. They should also be associated with genetic variants that have large effects, but how large an effect is necessary for a genetic variant to have practical utility is open to debate. If the effects are of the same magnitude as those observed for clinical phenotypes, similarly large but much harder to obtain consortium-based megasamples will be needed, which is likely to present a practical barrier.

In a series of papers, my colleagues and I extracted 17 endophenotypes from five electrophysiological protocols including spontaneous scalp-recorded electroencephalographic activity, brain event-related potentials in a visual stimulus discrimination task, an antisaccade eye tracking measure of inhibitory control, emotional modulation of the defensive startle eye-blink reflex, and sympathetic nervous system arousal indexed by electrodermal activation [2]. We used the same a priori analyses for all 17 endophenotypes, and published the results simultaneously so as to avoid the many problems believed to account for irreproducible scientific findings. We used an unscreened, epidemiological sample of over 4900 twin and family participants that was broadly representative of the Minnesota state population. Therefore, the results were not conditional on arbitrarily imposed inclusion or exclusion criteria, which can complicate replicability. We investigated common and rare variants and carried out empirically driven, discovery-based analyses, and also hypothesis-driven candidate gene and single nucleotide polymorphism (SNP) analyses.

‘SNP heritability’ was evaluated using genome-wide complex trait analysis, which confirmed that unrelated individuals who had the same endophenotype shared SNPs in common. From this, we can conclude that, in this study sample, the investigated endophenotypes were heritable, and that their genetic signal could be detected in the examined SNPs. Nevertheless, we were unable to corroborate any of the findings previously reported in the literature. The strongest effect size that we found was for antisaccade error and, even though undoubtedly inflated, the effect accounted for less than 1% of the variance in antisaccade inhibitory control. The P300 event-related potential—the positive brain wave deflection that occurs approximately 300 ms after an unexpected event—has been linked to over half a dozen different disorders and is one of the most studied and validated endophenotypes. The largest effect size that we found for P300 would require a sample of over 20,000 individuals to achieve genome-wide significance. We concluded that none of our endophenotypes were associated with genetic variants that had large effect sizes. Our endophenotypes proved to be polygenic complex traits, just like the clinical phenotypes with which they are associated. In this regard, they are much like genetic biomarkers associated with medically relevant conditions, such as cholesterol level, bone mineral density, body mass index, and heart rate [3].

With the exception of resting heart rate, which has received almost no attention as a possible endophenotype but which has been successfully associated with genetic variants by medical biomarker scientists, there is no psychiatric endophenotype that has widely accepted verified genetic variants [3]. We cannot say that such a finding will never emerge, but we can say that endlessly pursuing a small-sample, underpowered research strategy with the expectation that it will yield some sort of breakthrough genetic insight is unlikely to be successful. With sample sizes that number in the tens of thousands, we should be able to flesh out the genetic architecture of endophenotypes, but at a cost that is likely to be considerably greater than that associated with reliance on measures that are based on questionnaires and interviews.

## Recent studies utilizing endophenotypes and future prospects

Impaired social visual engagement is a prototypical endophenotype for autism spectrum disorder. It is assessed by monitoring ocular motion while participants view human interactions. The high heritability of this autistic feature has recently been demonstrated in twin toddlers, and 2-year-old children who fall within the autism spectrum have been found especially deficient in directing their gaze toward facial features that are important for social communication [4]. This finding suggests that it may be possible to identify children who are at high risk for autism who are particularly likely to have social communication deficits, thus possibly guiding early intervention strategies to lessen the impact of the deficit.

An undercapitalized strength of endophenotypes stems from their ability to index genetic susceptibility. Although more research is required to document how endophenotypes change with age, they have been used in longitudinal studies to predict the development of psychopathology over both short time intervals (e.g., [5]) and periods as long as 12 years (e.g., [6]). Prospectively following youths who are at ‘endophenotypic high disease risk’ would facilitate the tracking of psychopathology development, providing opportunity to identify the environmental potentiators and other risk factors that contribute to the unfolding of manifest pathology. Endophenotypes may also inform prognosis, and identify mechanistically relevant targets for intervention [7].

Although endophenotypes were originally envisioned to be disorder-specific, it is now widely recognized that psychiatric disorders are heterogeneous; comorbidity is the rule rather than the exception. It is thus not the case that everyone with a disorder can be expected to have the same endophenotype, nor are endophenotypes necessarily disorder-specific. Of 36 electrophysiological endophenotypes that were investigated [3], half were associated with three to seven different disorders (see also [8]), and most disorders have multiple endophenotypes. The fact that endophenotypes are transdiagnostic represents another underappreciated feature that can be used to develop insights into the nature of comorbidity and how genetic risk is shared across disorders or is specific to one. The comorbidity of Tourette syndrome and obsessive compulsive disorder (OCD) has been exploited to develop a novel endophenotype comprising obsessions with symmetry and ordering. In a large Tourette family study, symmetry was associated with a polygenic risk score for Tourette disorder, but not one for OCD, thus identifying genetic liability specific to Tourette that is not captured by the Diagnostic and Statistical Manual of the American Psychiatric Association (DSM). Using multiple endophenotypes that cut across psychotic disorder diagnoses, it has been possible to create clinically meaningful biotypes that have the potential to aid genetic discovery [9].

When psychopathology-related genetic variants are identified, we can determine the endophenotypes with which they are associated, with the hope that insights will be derived regarding how the genotype influences pathophysiology. As our understanding of the functional significance of verified genetic findings increases, it should be possible to use this genetic insight to develop improved endophenotypes that tap directly into pathophysiological processes, enhancing understanding of brain mechanisms and improving the identification of those with genetic liability. The *CACNA1C* calcium-channel-regulating gene has repeatedly emerged in association with severe psychiatric disorders, but the mechanisms through which it affects disorder risk are poorly understood. A series of knockout gene animal experiments in which *CACNA1C* is inactivated in forebrain glutamergic neurons found impairment in behavioral endophenotypes involving cognitive and social processing, thus highlighting how psychiatric endophenotypes can contribute insight into gene function [10].

## Conclusions

Although endophenotypes have not lived up to their promise in facilitating gene discovery for disease risk, they are nonetheless of significant value, with potential to index genetic liability, inform research on the development of psychopathology and prognosis, and contribute to our understanding of gene function and transdiagnostic etiology. Perhaps their greatest contribution since their introduction by Gottesman and Shields [1] half a century ago has been to shift the approach to how psychopathology research is profitably conducted, away from the expectation that DSM refinements are key to uncovering etiological insights, and toward the use of genetically informed laboratory measures to provide such understanding.

## Abbreviations

DSM: Diagnostic and Statistical Manual of the American Psychiatric Association
OCD: Obsessive compulsive disorder
SNP: Single nucleotide polymorphism
